# Posterior Reversible Encephalopathy Syndrome in the Context of McCune-Albright Syndrome: A Case Report

**DOI:** 10.7759/cureus.76261

**Published:** 2024-12-23

**Authors:** Kyriaki Astara, Christos Stenos, Konstantinos Kalafatakis, Maria Lypiridou, Georgios Stouraitis

**Affiliations:** 1 Department of Neurology, 417 Army Share Fund Hospital, Athens, GRC; 2 Department of Neurology, Errikos Ntynan Hospital Center, Athens, GRC; 3 Department of Neurology, Pammakaristos General Hospital, Athens, GRC; 4 Laboratory of Human-Computer Interaction, University of Ioannina, Arta, GRC

**Keywords:** acth-dependent cushing syndrome, mccune-albright syndrome, posterior reversible encephalopathy syndrome (pres), severe hypertension, subclinical hypothyroidism

## Abstract

Posterior reversible encephalopathy syndrome (PRES) is a clinical manifestation of various underlying causes, characterized by the combination of clinical and imaging findings associated with the posterior cerebral areas and relating to arterial hypertension and endothelial dysfunction. No association was made so far between PRES and McCune-Albright syndrome (MAS), a rare genetic disorder resulting in fibrous dysplasia.

A 33-year-old female with MAS was presented to the emergency department of the 417 Army Share Fund Hospital in Athens (Greece) after seizure activity with two episodes of ocular upward deviation and transient facial palsy, each lasting a few minutes, followed by a postictal phase. On arrival, vital sign measurements revealed sinus tachycardia and elevated systolic and diastolic blood pressure (177/102 mm Hg). A neurologic examination demonstrated irritability and confusion, complete blindness from the left eye, and progressive visual loss from the right due to compression of optic nerves by fibro-osseous tissue bilaterally. Neuroimaging revealed the symmetrical presence of signs of vasogenic edema and dysfunction of the posterior parts of the cerebral hemispheres and, in conjunction with the clinical manifestations, advocated the diagnosis of PRES. The patient was treated with systematic administration of anticonvulsants, antihypertensive agents, and nebivolol. Laboratory examination indicated the presence of Cushing syndrome. The patient was discharged afebrile, hemodynamically stable, and clinically improved.

MAS covers a spectrum of endocrine dysregulation, resulting in clinical manifestations of high variability. Even in cases of mild hypercortisolemia, it is vital to raise the clinical suspicion of CS, as its reverberations may occur with abrupt onset, like PRES.

## Introduction

Posterior reversible encephalopathy syndrome (PRES) is characterized by the combination of clinical and imaging findings associated with the posterior cerebral areas. Such manifestations involve visual defects, like hemianopsia, headache, seizures (focal or generalized), impaired consciousness, as well as signs of vasogenic edema with hyperintense T2-weighted or hypointense T1-weighted signals on magnetic resonance imaging (MRI), typically found symmetrically in the posterior subcortical regions [1]. As was first described by Hinchey et al. and updated throughout the last three decades, the condition is usually reversible [2]. Epidemiologically, PRES extends through all ages, with a peak incident reported in middle-aged adults with a preponderance of female patients [3]. The etiology of PRES fundamentally relates to arterial hypertension, while other factors that induce endothelial dysfunction have also been identified. Elevated blood pressure exceeds the upper autoregulatory limit, while endothelial dysfunction, caused by either endogenous (e.g., eclampsia) or exogenous (e.g., immunosuppressive agents) factors, leads to vasogenic edema [4]. The reason behind the predilection for the posterior areas remains to be elucidated, although theories support reduced autonomic regulation in the posterior vasculature of the brain.

In parallel, McCune-Albright syndrome (MAS) consists of a clinical pattern of fibrous dysplasia (FD), a benign fibro-osseous lesion related to abnormal bone development and replacement by fibrous tissue [5]. MAS is a rare genetic disorder, occurring in up to 1/1.000.000 cases, and is caused by a sporadic mutation in the GNAS gene on chromosome 20q13.3 [6]. MAS manifests typically with symptoms of FD (pain, recurrent fractures), café-au-lait skin spots (large maculae with irregular borders), and endocrine hyperactivity [7]. Here, we report a case of PRES in a hypertensive female patient with MAS.

## Case presentation

A 33-year-old woman with a history of MAS characterized by complete blindness from the left eye and progressive visual loss from the right due to compression of optic nerves by fibro-osseous tissue bilaterally, hypothyroidism, multiple fractures with osteotomies, and panic disorder presented to the emergency department of the 417 Army Share Fund Hospital in Athens (Greece) with suspected focal-onset seizures. Family members noticed a gradual deterioration in verbal responses and level of consciousness over the last six days, while the patient was showing unwillingness to engage in routine daily activities. On the day of the visit, the patient suffered two episodes of ocular upward deviation and transient facial palsy, each lasting a few minutes, followed by a postictal phase.

On arrival, vital sign measurements revealed a tachycardia of 105 bpm and elevated systolic and diastolic blood pressure (177/102 mm Hg), while temperature and oxygen saturation were normal. An electrocardiogram showed sinus tachycardia. A neurologic examination demonstrated irritability and confusion but no focal signs of acute onset. From the laboratory evaluation in the emergency department, the patient’s white blood cells were characterized by a neutrophil predominance (83%), indirect bilirubin was elevated (1.13 mg/dl), as well as alkaline phosphatase (311 IU/L), γ-glutamyl transferase (188 IU/L) (Table 1), and lactate dehydrogenase (339 IU/L).

**Table 1:** Basic laboratory examination MCHC: mean corpuscular hemoglobin concentration, MCV: mean corpuscular volume, Hgb: hemoglobulin, Hct: hematocrit, ESR: erythrocyte sedimentation rate, CRP: C-reactive protein, ALT: alanine transaminase, AST: aspartate transaminase, ALP: alkaline phosphatase, γGT: γ-glutamyltransferase, CPK: creatine-phosphate kinase, TIBC: total iron-binding capacity, INR: international normalized ratio, PT: prothrombin time, aPTT: activated partial thromboplastin time, LDL: low-density lipoprotein, HDL: high-density lipoprotein, Ca: calcium, P: phosphorus, K⁺: potassium, Na⁺: sodium, Mg²⁺: magnesium, Cl⁻: chloride, CPK: creatine phosphokinase, LDH: lactate dehydrogenase, Fe: iron, TIBC: total iron-binding capacity, CA: cancer antigen

Laboratory test	Value	Reference value
White blood cell count	7.5 K/μL	4.0-10.0 K/μL
Neutrophil %	83%	40-75%
Lymphocyte %	10.7%	20-45%
Red blood cell count	5.0 M/μL	4.5-4.8 M/μL
MCHC	30.0 pg	26.0-32.0 pg
MCV	91.2 fL	79.0-100 fL
Hgb	15 g/dL	13.7-17.9 g/dL (men), 11.6-15.5 g/dL (women)
Hct	45.6%	36-48%
Platelets	241 K/μL	140-450 K/μL
INR	0.99	-
PT	10.9 sec	-
aPTT	24.6 sec	26.0-36.0 sec
ESR	16 mm/h	<28 mm/h
CRP	0.1 mg/dL	<0.5 mg/dL
Total bilirubin	1.57 mg/dl	0.20-1.10 mg/dl
Direct bilirubin	0.44 mg/dl	0.10-0.50 mg/dl
ALT	27 U/L	5-40 U/L
AST	49 U/L	5-40 U/L
ALP	311 IU/L	40-140 IU/L
γGT	188 IU/L	10-50 IU/L
Total cholesterol	190 mg/dL	125-200 mg/dL
LDL	111 mg/dL	100-160 mg/dL
HDL	56 mg/dL	>40 mg/dL
Triglycerides	113 mg/dL	35-150 mg/dL
Creatinine	0.5 μmol/L	0.7-1.4 μmol/L
Urea	20 mg/dl	10-55 mg/dl
Ca	8.9 mg/dL	8.4-10.2 mg/dL
P	2.1 mg/dL	2.5-4.5 mg/dL
K+	3.7 mmol/L	3.5-5.1 mmol/L
Na+	142 mmol/L	136-150 mmol/L
Mg2+	1.85 mg/dL	1.62-2.62 mg/dL
Cl−	103 mmol/L	98-107 mmol/L
Glucose	103 mg/dl	70-110 mg/dl
Amylase	87 IU/L	25-125 IU/L
CPK	84 IU/L	24-170 IU/L
LDH	339 IU/L	100-220 IU/L
Total protein	5.30 g/dL	6.40-8.30 g/dL
Albumin	3.20 g/dL	3.40-5.0 g/dL
Fe	80 mg/dL	50-170 mg/dL
TIBC	246 mg/dL	250-450 mg/dL
Ferritin	149.2 mg/L	4.63-204 mg/L
Vitamin B12	343 pg/mL	179-1162 pg/mL
a-Fetoprotein	6.0 ng/ml	0.0-5.0 ng/ml
CA 19-9 antigen	14.1 U/ml	0.0-37.0 U/ml
CA 153 antigen	20.7 U/ml	0.0-36.0 U/ml
CA 125 antigen	15.7 U/ml	0.0-31.0 U/ml

Chest radiograph displayed severe deformity of the bones of the chest wall with the involvement of ribs, both clavicles, humerus, and thoracic and lumbar spine (suggesting possibly past pathological fractures), evidence of ground-glass matrix in the lung parenchyma, as well as enlargement of a cardiac silhouette with severe decrease of lung volumes. An emergency brain computed tomography scan was negative for intracranial hemorrhage and did not show any evidence of ischemia. The patient was admitted to the neurology department for further evaluation and management. She was given intravenous loading with diazepam and valproic acid to prevent the recurrence of seizure activity and amlodipine for blood pressure regulation.

**Figure 1:**
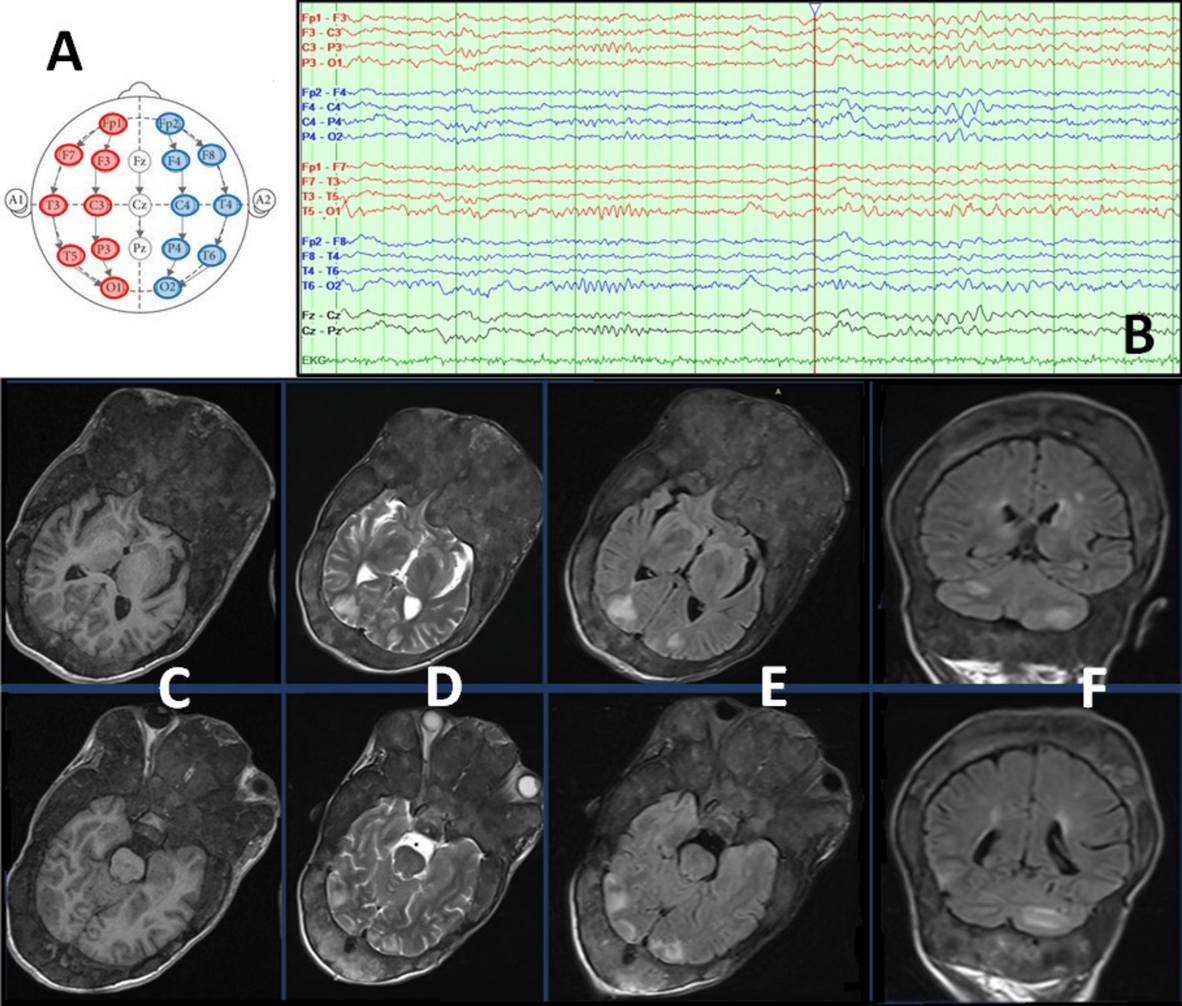
Electroencephalography and brain imaging (A) The International 10–20 system was followed for the placement of scalp electrodes during the EEG exam. (B) No epileptiform activity was observed. Note the predominance of low-frequency, high-amplitude (delta and theta) waves, primarily corresponding to the posterior scalp electrodes, indicating reduced neural activity. No lateralization was observed. (C-E) Axial brain MRI images: T1-weighted (C), T2-weighted (D), and T2-weighted cerebrospinal fluid-suppressed (FLAIR) (E) at the level of the interthalamic adhesion (top) and the interpeduncular cistern (bottom). Areas of hypointense T1 and hyperintense T2 signals are evident in the parieto-occipital areas bilaterally, along with compression of the optic nerves by fibro-osseous tissue bilaterally. (F) Coronal FLAIR brain MRI at the level of the cerebellar vermis and vein of Galen (top) and the posterior part of the straight sinus near the confluence of sinuses (bottom). Areas of hyperintense T2 signals are observed in the cerebellar hemispheres bilaterally, as well as in the centrum semiovale and corona radiata. EEG: electroencephalogram, MRI: magnetic resonance imaging, FLAIR: fluid-attenuated inversion recovery

During her hospitalization, she underwent electroencephalogram (EEG), brain MRI, and magnetic resonance angiography (MRA) of intracranial and extracranial vessels with arterial and venous time. EEG displayed an encephalopathic chart with slowing of the posterior dominant rhythm, followed by a gradual symmetrical deceleration with the appearance of theta and, rarely, delta activity without hemispheric lateralization (Figure 1A-1B). MRI findings showed hypointense lesions in T1-weighted and hyperintense in T2-weighted and fluid-attenuated inversion recovery (FLAIR) images, extending principally in the parieto-occipital areas bilaterally and to a lesser extent in both cerebellar hemispheres (Figure 1C-1F). The possibility of acquiring gadolinium-enhanced images was opted out due to the unclear history of allergies. No pathological findings were noted in MRA, and magnetic resonance venography showed normal imaging of dural venous sinuses and cerebral veins.

Overall, evaluation of the imaging findings (symmetrical presence of signs of vasogenic edema in the posterior parts of the cerebral hemispheres) in conjunction with the clinical manifestations advocated the diagnosis of PRES.

After the initial systematic administration of anti-seizure medication and antihypertensive agents, altered mental status and arterial hypertension gradually resolved, while tachycardia remained until a beta-blocker was added to the patient's treatment plan. Further thorough laboratory examination indicated the abnormal release of cortisol, as the urine-free cortisol value was 265 mcg/24 hours, and the low dose dexamethasone suppression test was 1005 nmol/L (normal: <50 nmol/L). There is also a suspicion of subclinical hyperthyroidism (Table 2). After 10 days of hospitalization, the patient was discharged afebrile and hemodynamically stable, restoring her level of consciousness and communication to their baseline before being admitted to the hospital.

**Table 2:** Hormonal laboratory examination Thyroid function tests indicated only slightly decreased thyrotropin (TSH), a finding consistent with subclinical hyperthyroidism. It should be noted that the patient had previously undergone radioiodine therapy due to more pronounced hyperthyroidism. It should also be noted that a recent review paper, which analyzed existing MAS cases complicated by hyperthyroidism, concluded that radioiodine therapy is possibly expected to lead to a recurrence of hyperthyroidism [8]. CS was diagnosed based on free cortisol levels and the low-dose dexamethasone suppression test. Cortisol levels were three times higher than normal values and remained high after the nightly administration of 1 mg of dexamethasone. T3: triiodothyronine, T4: levothyroxine, TSH: thyroid-stimulating hormone, MAS: McCune-Albright syndrome, CS: Cushing syndrome

Laboratory test	Value	Reference value
Free T3	300 pg/mL	260-480 pg/mL
Free T4	1.1 ng/dl	0.7-1.53 ng/dl
TSH	0.40 μU/ml	0.5-5.0 μU/ml
Urine-free cortisol	365 μg/24h	<100 μg/24h
Low-dose dexamethasone suppression test	1005 nmol/L	<50 nmol/L

## Discussion

We presented the case of a 33-year-old female patient with MAS who was diagnosed with PRES. Various endocrinopathies accompany MAS. For instance, gonadotropin hormone-releasing hormone-independent precocious puberty is the most common endocrinopathy and exhibits suppressed LH and FSH, as well as elevated levels of testosterone and estradiol. Other endocrinopathies include hyperthyroidism, acromegaly, and Cushing syndrome (CS) [9]. Due to the osseous deformities, compression of cranial nerves may occur, which is the typical manifestation.

Due to the manifold expression of MAS, hypertension is expected to accompany its clinical presentation in the context of hypercortisolism, or CS. However, to our knowledge, only one natural history study has investigated the prevalence of CS in MAS patients, estimated at approximately 22% [10]. In this sub-group of MAS patients with CS, hypertension was recorded in 33% of them. In the present case report, our patient was presented with previously undiagnosed hypertension grade 3. On another note, the development of PRES in the context of CS has only been highlighted in the last decade [11].

When mean arterial pressure sustainably rises >160 mmHg, the upper limit of cerebral blood flow autoregulation is exceeded. The myogenic tone is lost, forcing cerebral vasodilation, which drops cerebrovascular resistance and produces hyperperfusion. In this state, hydrostatic pressure increases, and cerebral endothelium leakage leads to vasogenic edema formation. This integrates the most prevalent pathophysiologic noxious milieu in which PRES may emerge [12,13].

PRES, like MAS, may occur with various neurological symptoms, depending on the etiology and the anatomical region. Onset is usually acute within a few hours but may follow a rather subacute course. Clinical manifestations included visual disturbances, blurred vision, hemianopsia, and even cortical blindness, signs of encephalopathy with altered mental status, and other less specific symptoms like headache, nausea, and vomiting [14]. Seizures, particularly generalized, emerge frequently and can even end up in nonconvulsive status epilepticus [15]. In our case, the patient was admitted to our hospital due to focal onset seizures.

MRI findings in our patient were consistent with PRES. Signal characteristics of affected areas usually reflect vasogenic edema. In T1-weighted images, affected regions are presented as hypointense lesions, and after gadolinium administration, a variable enhancement in either a leptomeningeal or cortical pattern may become obvious. In T2-weighted images, the signal is hyperintense. Diffusion-weighted imaging is usually normal but may sometimes exhibit hyperintensities due to edema (T2 shine-through) or true restricted diffusion, while gradient echo/susceptibility-weighted imaging may show hemorrhages (including microhemorrhages). Angiography may show patterns of vasculopathy with vessel irregularity consistent with focal vasoconstrictions/vasodilatation and diffuse vasoconstriction [16].

Bridging MAS with PRES pathophysiology, the GNAS locus has been implicated in hypertension by Jia et al., who identified polymorphisms of the GNAS1 locus associated with hypertension and response to β-blockers [17]. Additionally, in the context of CS, cortisol may directly induce endothelial dysfunction by decreasing nitric oxide, a major vasodilatory compound, as well as increasing oxidative stress [18].

To the best of our knowledge, this is the first case report of PRES [19], caused by CS, developed in the context of MAS.

## Conclusions

PRES is a neurological disorder developed by rapidly increasing arterial blood pressure and/or endothelial dysfunction. Any systematic condition associated with these two pathophysiological mechanisms could lead to PRES. CS has been associated with PRES since hypercortisolism is related to refractory hypertension, while increased cortisol levels might also cause endothelial dysfunction. CS has also been developed in the context of MAS.
